# Alpinetin exerts anti-colitis efficacy by activating AhR, regulating miR-302/DNMT-1/CREB signals, and therefore promoting Treg differentiation

**DOI:** 10.1038/s41419-018-0814-4

**Published:** 2018-08-30

**Authors:** Qi Lv, Can Shi, Simiao Qiao, Na Cao, Chunge Guan, Yue Dai, Zhifeng Wei

**Affiliations:** Department of Pharmacology of Chinese Materia Medica, School of Traditional Chinese Pharmacy, China Pharmaceutical University, 24 Tong Jia Xiang, Nanjing, 210009 China

## Abstract

Alpinetin, a flavonoid compound extracted from the seeds of *Alpinia katsumadai* Hayata, has been demonstrated to exert massive biological properties. This study aimed to evaluate the effect of alpinetin on dextran sulfate sodium (DSS)-induced colitis, and elucidate the potential mechanisms. Alpinetin significantly alleviated colitis in mice, accompanied with restored Th17/Treg balance in colons. In vitro, alpinetin directly promoted Treg differentiation but exerted little effect on Th17 differentiation, and the action was in an aryl hydrocarbon receptor (AhR)-dependent manner. It acted as a potential AhR activator, evidenced by increased expression of CYP1A1, dissociation of AhR/HSP90 complexes, AhR nuclear translocation, XRE-driven luciferase reporter gene and DNA-binding activity of AhR/ARNT/XRE in T cells. Furthermore, alpinetin significantly promoted expression of miR-302 but not others, and restrained expression of DNMT-1 and methylation level of Foxp3 promoter region in CD4^+^ T cells and colons of colitis mice. However, the association of CREB and Foxp3 promoter region but not expression, nuclear translocation and DNA-binding activity of CREB was up-regulated by alpinetin in CD4^+^ T cells. The relationship of alpinetin-adjusted AhR activation, expressions of miR-302 and DNMT-1, association of CREB and Foxp3 promoter region, and Treg differentiation was confirmed by using CH223191, siAhR, miR-302 inhibitor and pcDNA3.1(+)-mDNMT-1. Finally, CH223191 abolished the amelioration of alpinetin on colitis, induction of Treg cells and regulation of miR-302/DNMT-1/CREB signals in colons of colitis mice. In conclusion, alpinetin ameliorated colitis in mice *via* activating AhR, regulating miR-302/DNMT-1/CREB signals, therefore promoting Treg differentiation.

## Introduction

Ulcerative colitis (UC) is a chronic non-specific inflammatory disease, and mainly affects rectum and colon. Its pathogenesis is still unclear, genetic, infectious, immunological, environmental factors and intestinal dysbiosis have been identified and occupy key positions^1^. Recently, researchers believe that the balance between Th17 and Treg cells controls occurrence, development and severity of UC^2^. In colonic mucosa and peripheral blood of UC patients, percentages of Th17 cells significantly increase, which is closely related to the disease activity and severity^3^. In addition, IL-17^-/-^ mice are more resistant to dextran sulfate sodium (DSS)-induced colitis and present higher survival ratio, lower disease activity index (DAI) scores, and improved-pathological changes in colons^4^. However, percentages of Treg cells decrease in peripheral blood of UC patients, and Treg cells possess the ability to prevent the progress of colitis in mice^5,6^.

Aryl hydrocarbon receptor (AhR), a ligand-activated transcription factor, belongs to the basic-helix-loop-helix/Per-Arnt-Sim (bHLH/PAS) family. It can bind with both endogenous and exogenous ligands, and then regulates differentiation of multiple T cells^7^. 2, 3, 7, 8-tetrachlorodibenzo-p-dioxin (TCDD) inhibits the differentiation of CD4^+^ T cell into Th1, Th2 and Th17 cells, while inducing Foxp3-positive Treg cells^8^. In mice with DSS-induced colitis, knockout of AhR obviously increases the disease severity^9^. However, TCDD and tryptophan metabolite kynurenine can ameliorate the development of colitis in mice^10,11^. Therefore, therapeutic approach targeting at activating AhR and recovering the balance of Th17/Treg will be intriguing.

The seed of *Alpinia katsumadai* Hayata has been used to treat digestive system-related diseases in china for thousands of years. It contains a series of components, such as flavones and volatile oils. The total flavone components have previously been demonstrated to be a potential therapeutic agent for inflammation and immunity-related diseases. Alpinetin, the main flavonoid in *Alpinia katsumadai* Hayata, is proven to be able to prevent expressions of TNF-α, IL-6 and IL-1β in LPS-stimulated THP-1 cells by inhibiting activation of NF-κB and MAPK signaling pathway, and markedly regulate ratio of CD4^+^/CD8^+^ in ConA-induced splenocytes in vitro^12,13^. Furthermore, the results in vivo demonstrate that alpinetin markedly attenuates DSS-induced acute colitis through TLR4 and NLRP3 pathways^14^. Interestingly, multiple kinds of flavonoids possess the ability to regulate Th17/Treg balance (baicalin, epigallocatechin gallate, *etc*) and activate AhR (kaempferol, quercetin, *etc*)^15–17^. Therefore, we worked to investigate the anti-colitis efficacy of alpinetin, and explore the key mechanisms from angle of activating AhR and recovering Th17/Treg balance.

## Results

### Alpinetin attenuated DSS-induced colitis in mice

**Fig. 1:**
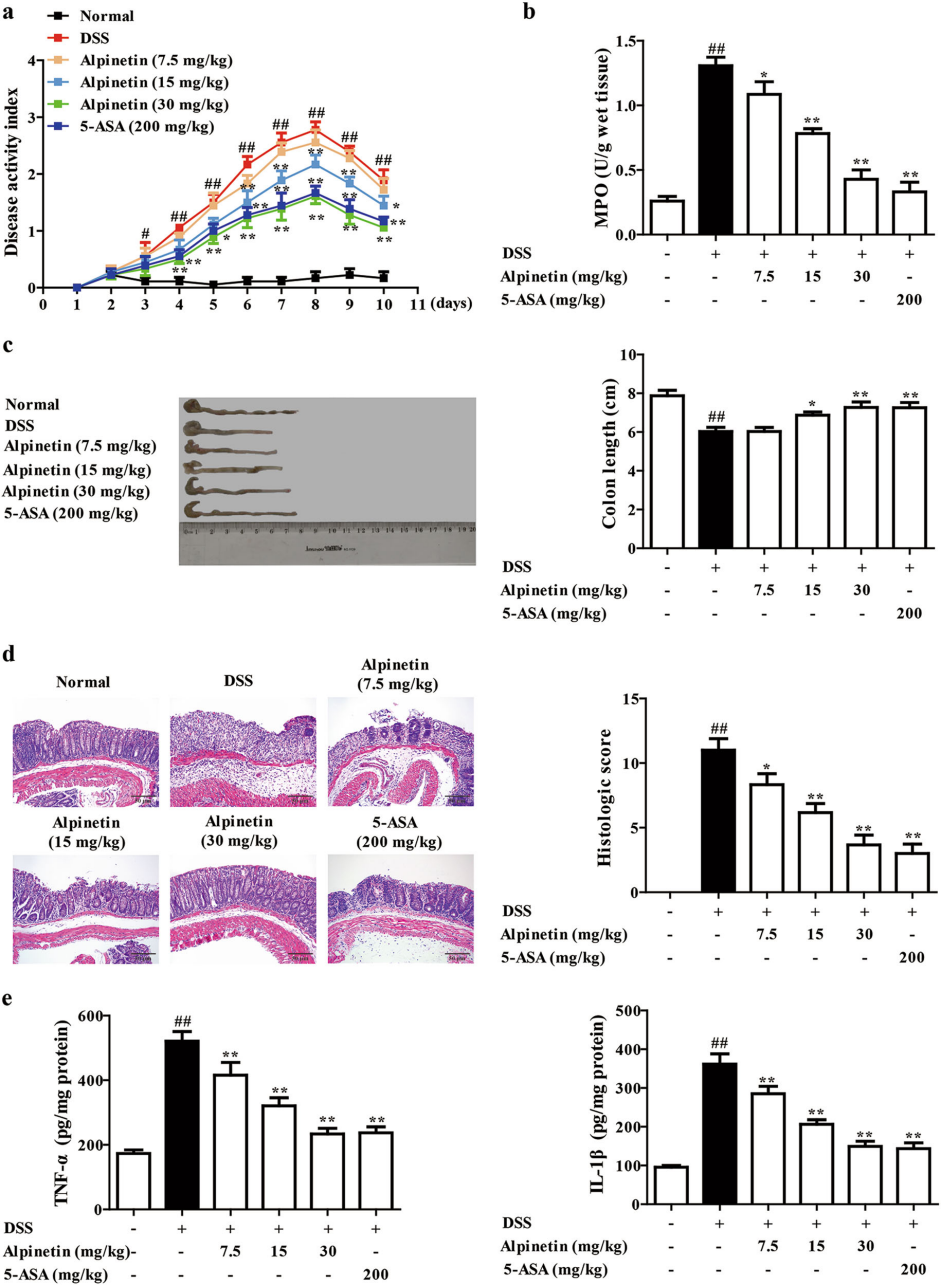
Effect of alpinetin on DSS-induced colitis in mice. Mice were orally administrated of 2.5% DSS for 7 days, and followed by sterile distilled water alone for another 3 days. The alpinetin (7.5, 15, 30 mg/kg) and 5-aminosalicylic acid (5-ASA, 200 mg/kg) were orally administered daily for consecutive 10 days. Then, mice were sacrificed, and colons were collected on day 10. **a** Disease activity index (DAI) scores. **b** The activity of myeloperoxidase (MPO) in colons. **c** The colon length. **d** The histological changes were detected by using hematoxylin and eosin (H&E) staining (Scale bar: 50 μm). **e** The protein levels of TNF-α and IL-1β in colons were detected by using ELISA. The data were presented as means ± S.E.M. of six mice in each group. ^#^*P* *<* 0.05, ^##^*P* *<* 0.01 vs. the group without any treatment; ^*^P *<* 0.05 and ^**^P *<* 0.01 vs. DSS group

To determine the therapeutic potential of alpinetin on colitis, mice were challenged with 2.5% DSS. As expected, mice in DSS group exerted dramatic increased DAI scores, and oral administration of alpinetin (15, 30 mg/kg) showed significant reduction (Fig. 1a). Furthermore, alpinetin (7.5, 15, 30 mg/kg) dose-dependently reduced the myeloperoxidase (MPO) activity, which is a marker of neutrophil infiltration (Fig. 1b). The shortening of colon length was also prevented (Fig. 1c). Results of hematoxylin and eosin (H&E) staining in colons of colitis mice further revealed obvious pathological changes, including severe damage in the surface epithelium, a pronounced decrease in the number of crypts and infiltration of inflammatory cells, and alpinetin (7.5, 15, 30 mg/kg) displayed significant improvement in a dose-dependent manner (Fig. 1d).

In addition, mice with DSS-induced colitis exhibit a cytokine profile similar to colitis patients, including overproduction of proinflammatory cytokines TNF-α and IL-1β. In this study, protein levels of TNF-α and IL-1β in colons of colitis mice were markedly increased, and alpinetin (7.5, 15, 30 mg/kg) exerted obvious inhibition (Fig. 1e).

### Alpinetin restored the Th17/Treg balance in colons of colitis mice

**Fig. 2:**
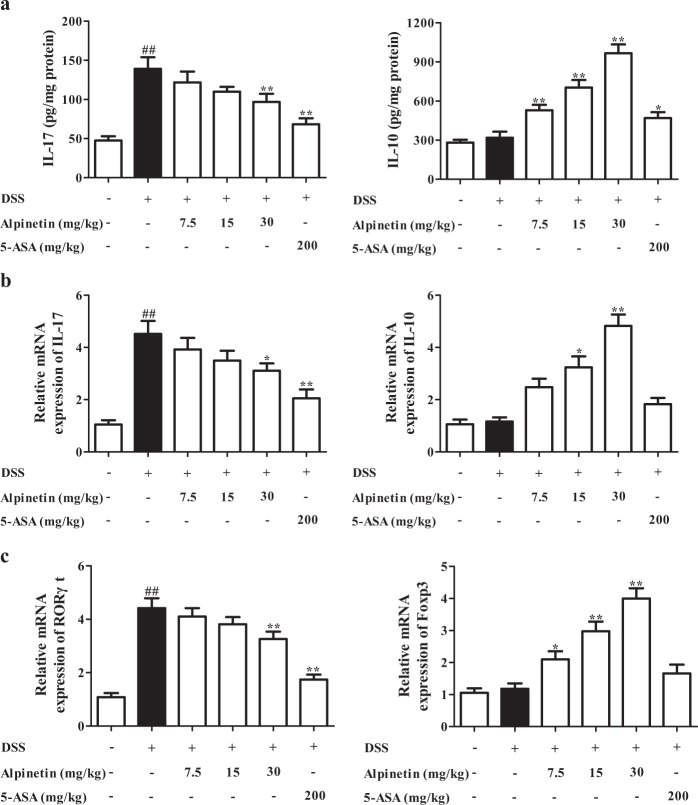

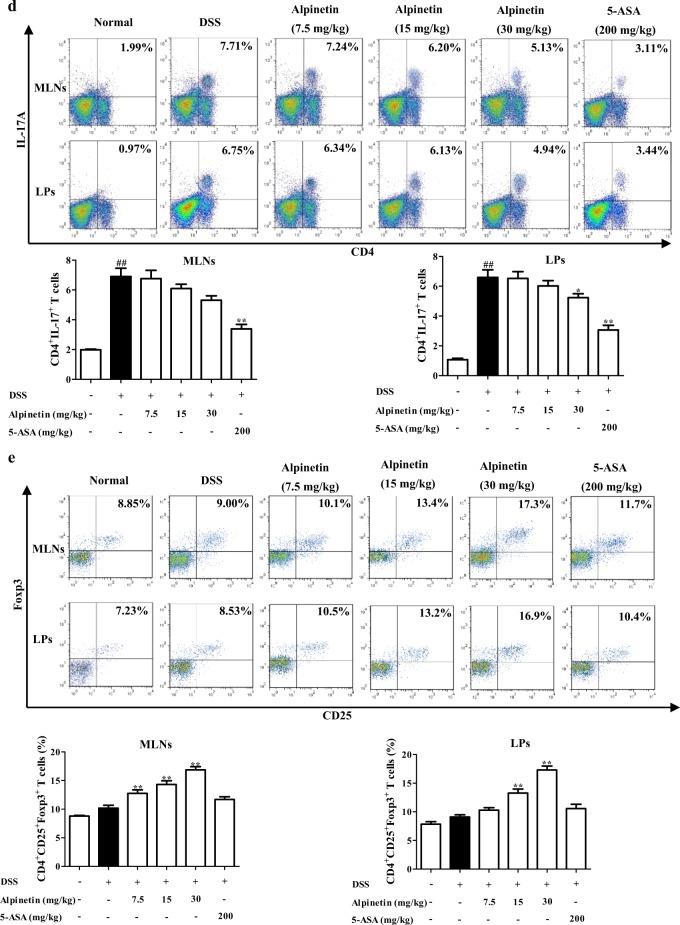

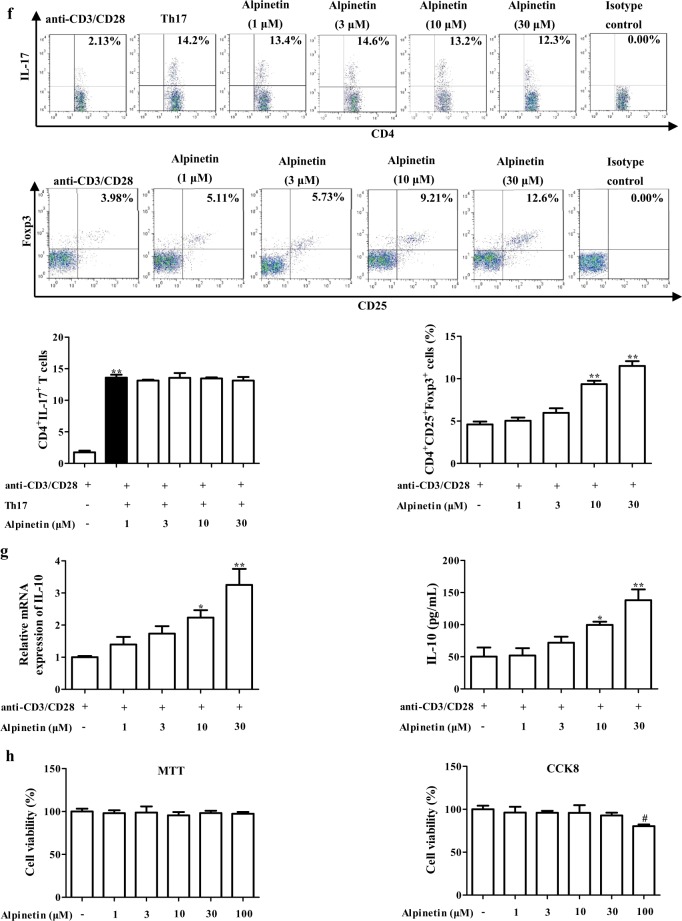
Effect of alpinetin on the imbalance of Th17/Treg. **a**–**d** Mice were orally administrated of 2.5% DSS for 7 days, and followed by sterile distilled water alone for another 3 days. The alpinetin (7.5, 15, 30 mg/kg) and 5-aminosalicylic acid (5-ASA, 200 mg/kg) were orally administered daily for consecutive 10 days. Then, mice were sacrificed, and colons were collected. The protein levels of IL-17 and IL-10 in colons were detected by using ELISA (**a**); the mRNA levels of IL-17, IL-10, RORγt and Foxp3 were detected by using Q-PCR assay (**b**, **c**); the percentages of Th17 cells and Treg cells in mesenteric lymph nodes (MLNs) and colonic lamina proprias (LPs) were detected by using flow cytometry assay (**d**, **e**). **f** CD4^+^ T cells were treated with anti-CD3/CD28 (2 μg/mL), rhTGF-β1 (5 ng/mL), rmIL-6 (20 ng/mL), rmIL-2 (300 IU/mL) and alpinetin (1, 3, 10, 30 μM) for 72 h, percentages of Th17 cells were detected by using flow cytometry assay. In addition, CD4^+^ T cells were treated with anti-CD3/CD28 (2 μg/mL) and alpinetin (1, 3, 10, 30 μM) for 72 h, percentages of Treg cells were detected by using flow cytometry assay. **g** CD4^+^ T cells were treated with anti-CD3/CD28 (2 μg/mL) and alpinetin (1, 3, 10, 30 μM) for 72 h, mRNA and protein levels of IL-10 were detected by using Q-PCR and ELISA, respectively. **h** CD4^+^ T cells were cultured with alpinetin (1, 3, 10, 30, 100 μM) for 72 h, the viability and proliferation of CD4^+^ T cells were detected by using MTT and CCK8 assays, respectively. The data were presented as means ± S.E.M. of three independent experiments or six mice in each group. ^##^*P* *<* 0.01 *vs*. the group without any treatment; ^*^P *<* 0.05 and ^**^P *<* 0.01 vs. DSS group or anti-CD3/CD28 group

The imbalance of Th17/Treg is a crucial event for the occurrence and development of UC, and multiple kinds of flavonoids isolated from plants such as baicalin and epigallocatechin gallate showed significant recovery of Th17/Treg balance^15,16^. Alpinetin (15, 30 mg/kg) significantly up-regulated levels of IL-10 and Foxp3 in colons of colitis mice. However, it only downregulated levels of IL-17 and RORγt at the dose of 30 mg/kg, and the strength was lower. The similar results were obtained from the proportions of Treg cells and Th17 cells in mesenteric lymph nodes (MLNs) and colonic lamina proprias (LPs) (Fig. 2a, e).

### Alpinetin could directly regulate the differentiation of Treg but not Th17 cells

To further confirm the above-mentioned results, CD4^+^ T cells of MLNs were isolated and prepared, and models of Th17 and Treg differentiation were established. Alpinetin (10, 30 μM) concentration-dependently promoted Treg differentiation, and the action was not associated with the participation of TGF-β. In addition, alpinetin (1, 3, 10, 30 μM) had slight effect on Th17 differentiation (Fig. 2f–g).

To exclude interference of alpinetin on cell viability and proliferation, MTT and CCK8 experiments were performed. As compared with the normal group, the viability and proliferation of CD4^+^ T cells was not affected by alpinetin (1, 3, 10, 30 μM) (Fig. 2h).

### AhR participated in alpinetin-promoted Treg differentiation

**Fig. 3:**
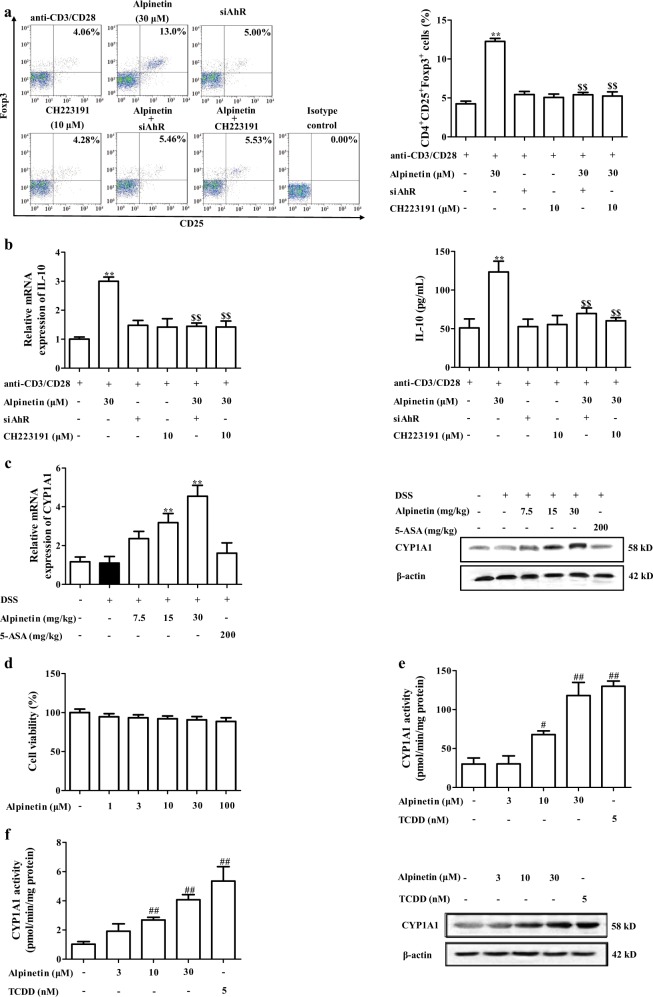

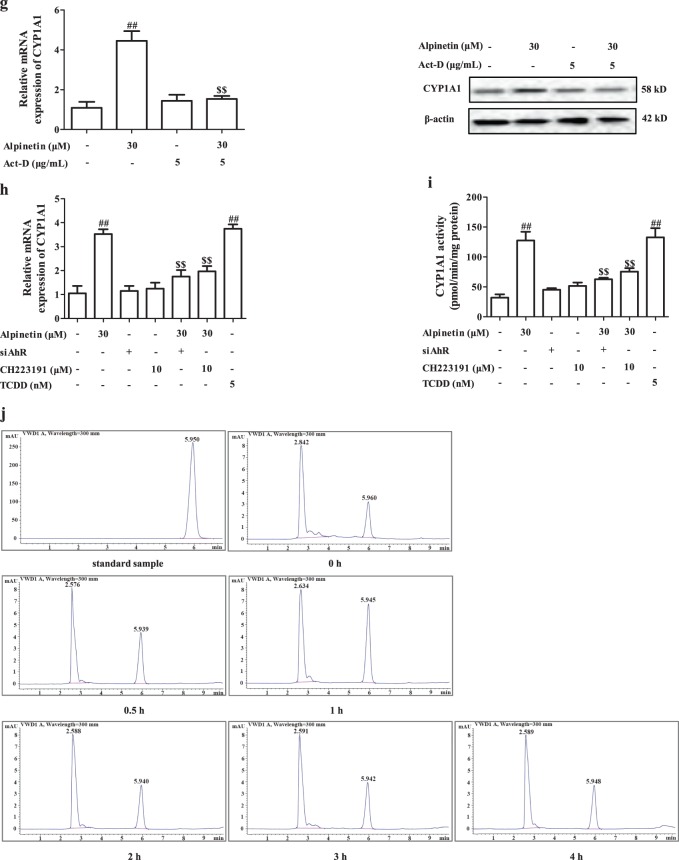

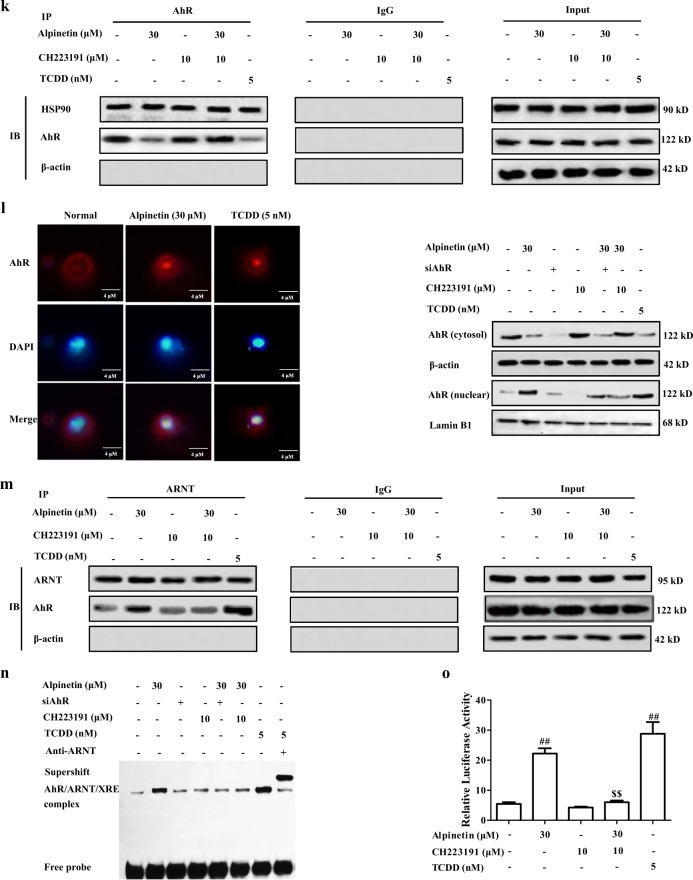
AhR played a key role in alpinetin-promoted Treg differentiation. **a**, **b** CD4^+^ T cells were treated with anti-CD3/CD28 (2 μg/mL), alpinetin (30 μM), siAhR, CH223191 (10 μM), siAhR + alpinetin, CH223191 + alpinetin for indicated times. At 72 h, percentages of Treg cells were detected by using flow cytometry assay (**a**); at 72 h, mRNA and protein levels of IL-10 were detected by using Q-PCR and ELISA, respectively (**b**). **c** Mice were subjected to colitis, and orally administered of alpinetin (7.5, 15, 30 mg/kg) and 5-aminosalicylic acid (5-ASA, 200 mg/kg). On day 10, the colons were collected. The mRNA and protein levels of CYP1A1 in colons of colitis mice were detected by using Q-PCR and western blotting assays, respectively. **d** EL-4 cells were treated with alpinetin (1, 3, 10, 30, 100 μM) for 24 h, cell viability was detected by using MTT assay. **e**, **f** EL-4 cells were treated with alpinetin (3, 10, 30 μM) and TCDD (5 nM) for 24 h, the activity, mRNA and protein levels of CYP1A1 were detected by using a commercial kit, Q-PCR and western blotting assays, respectively. **g** EL-4 cells were treated with Act-D (5 μg/mL), alpinetin (30 μM) and Act-D + alpinetin for 24 h, mRNA and protein levels of CYP1A1 were detected by using Q-PCR and western blotting assays, respectively. **h**, **i** EL-4 cells were treated with alpinetin (30 μM), siAhR, CH223191 (10 μM), siAhR + alpinetin, CH223191 + alpinetin and TCDD (5 nM) for 24 h, mRNA level and activity of CYP1A1 were detected by using Q-PCR assay and a kit. **j** EL-4 cells were treated with alpinetin (30 μM) for 0, 0.5, 1, 2, 3 and 4 h, and then harvested and lysed. The endocytosis of alpinetin was detected by using HPLC assay. **k**–**o** EL-4 cells were treated with alpinetin (30 μM), TCDD (5 nM), siAhR, CH223191 (10 μM), siAhR + alpinetin or CH223191 + alpinetin for 24 h. The association of HSP90 and AhR was detected by using Co-immunoprecipitation assay (**k**); the nuclear translocation of AhR was detected by using immunofluorescence and western blotting assays (Scale bar: 4 μm) (**l**); the association of AhR and ARNT was detected by using Co-immunoprecipitation assay (**m**); the DNA-binding activity of AhR/ARNT/XRE was detected by using EMSA assay (**n**); the activity of XRE-driven luciferase reporter gene was detected by using luciferase report gene assay (**o**). The data were presented as means ± S.E.M. of three independent experiments or six mice in each group. ^#^*P* *<* 0.05 and ^##^*P* *<* 0.01 vs. the group without any treatment; ^*^P *<* 0.05 and ^**^P *<* 0.01 vs. DSS group or anti-CD3/CD28 group; ^$$^*P* *<* 0.01 *vs*. alpinetin (30 μM) group

AhR, a ligand-dependent transcription factor, belongs to bHLH/PAS family. Recently, it has been demonstrated with well regulation of Th17/Treg balance, and multiple flavonoids such as baicalein, luteolin and kaempferol are natural ligands for AhR^17^. At present, siAhR and AhR antagonist CH223191 (10 μM) almost completely abolished alpinetin-promoted Treg differentiation (Fig. 3a, b). In addition, alpinetin (15, 30 mg/kg) obviously increased the mRNA and protein expressions of AhR target gene CYP1A1 in colons of colitis mice (Fig. 3c). All these data indicated that alpinetin-promoted Treg differentiation was mediated by AhR.

### Alpinetin could activate AhR in T cells

Subsequently, we explored the direct activation of AhR by alpinetin in EL-4 cells. At concentrations of 1, 3, 10, 30, 100 μM, alpinetin showed little effect on EL-4 cells viability (Fig. 3d). To determine whether the increase of CYP1A1 expression in response to alpinetin was a result of de novo RNA synthesis, EL-4 cells were pre-incubated with RNA synthesis inhibitor actinomycin D (Act-D). As expected, alpinetin-promoted expression of CYP1A1 was abolished by Act-D, indicating a requirement for de novo RNA synthesis. Furthermore, siAhR and CH223191 (10 μM) almost offset alpinetin-mediated increase of CYP1A1 expression and ethoxyresorufin-O-deethylase (EROD) activity in EL-4 cells (Fig. 3e–i). All these results indicated that alpinetin-promoted expression of CYP1A1 was dependent on RNA synthesis and AhR.

AhR locates at cytoplasm, and endoctoysis is a necessary process for alpinetin to activate AhR. EL-4 cells were treated with alpinetin (30 μM) for 0.5, 1, 2, 3 and 4 h, repeated freezing and thawing, and high performance liquid chromatography (HPLC) assay was performed. As shown in Fig. 3j, we could demonstrate the existence of alpinetin at the intracellular, and the amount was highest at 1 h. Furthermore, dissociation of AhR/HSP90 complexes, nuclear translocation of AhR, association of AhR/ARNT complexes, binding activity of AhR/ARNT/xenobiotic response elements (XRE) and XRE reporter activity were also promoted by alpinetin. In addition, when associated with siAhR or CH223191, the action of alpinetin disappeared (Fig. 3k–o). Taken together, alpinetin could activate AhR in EL-4 cells.

### The miR-302 played a key role in alpinetin-induced Treg differentiation after activating AhR

**Fig. 4:**
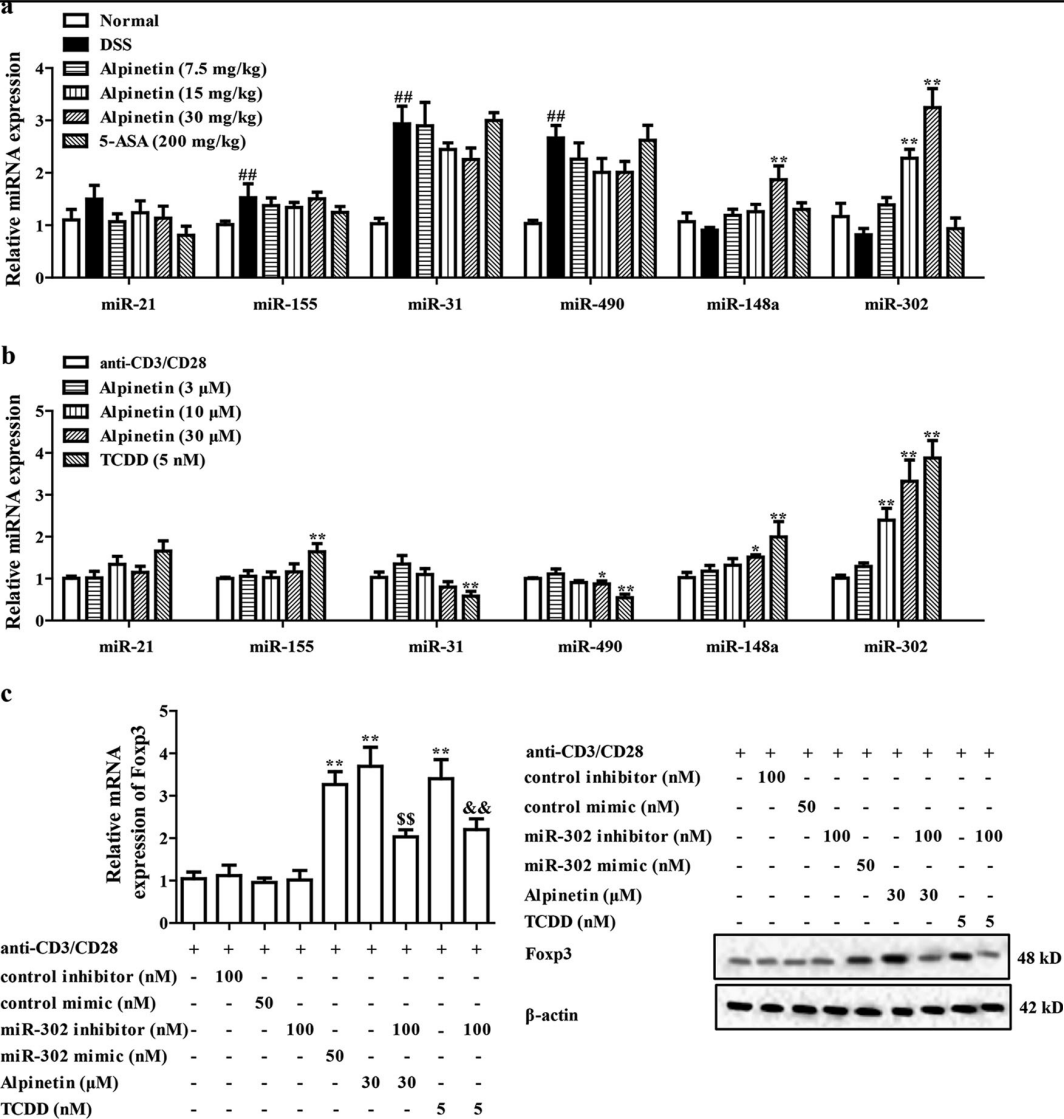

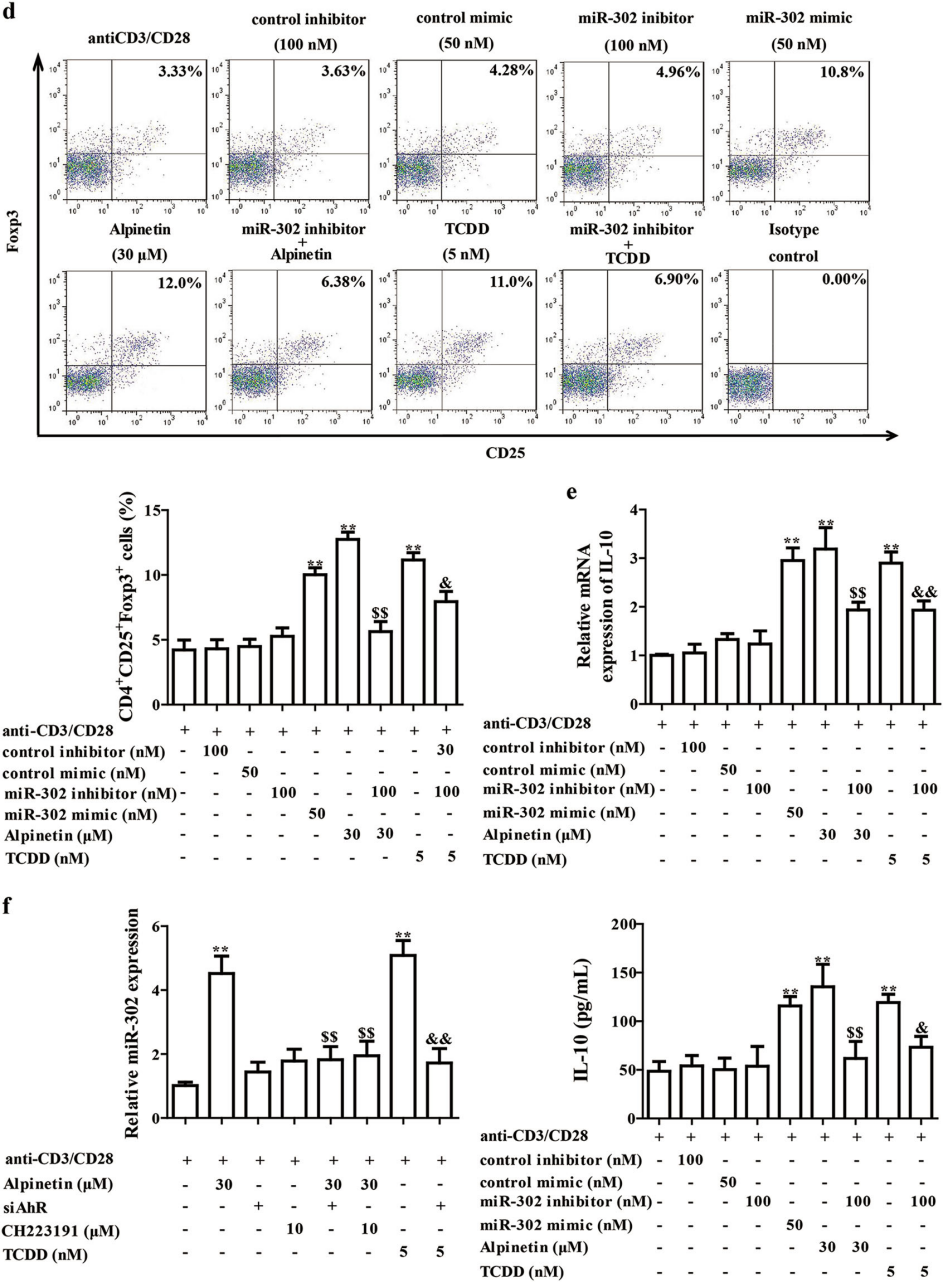
The miR-302 participated in alpinetin-promoted Treg differentiation after activating AhR. **a** Mice were subjected to colitis, and orally administered of alpinetin (7.5, 15, 30 mg/kg) and 5-aminosalicylic acid (5-ASA, 200 mg/kg). On day 10, the colons were collected. The expressions of miR-21, miR-155, miR-31, miR-490, miR-148a and miR-302 were detected by using Q-PCR assay. **b** CD4^+^ T cells were treated with anti-CD3/CD28 (2 μg/mL), alpinetin (3, 10, 30 μM) and TCDD (5 nM) for 48 h, expressions of miR-21, miR-155, miR-31, miR-490, miR-148a and miR-302 were detected by using Q-PCR assay. **c**–**e** CD4^+^ T cells were treated with anti-CD3/CD28 (2 μg/mL), alpinetin (30 μM), miR-302 mimic (50 nM), miR-302 inhibitor (100 nM), miR-302 inhibitor + alpinetin, TCDD (5 nM) and miR-302 inhibitor + TCDD for indicated time intervals. At 48 h, mRNA and protein levels of Foxp3 were detected by using Q-PCR and western blotting assays, respectively (**c**); at 72 h, percentages of Treg cells were detected by using flow cytometry assay (**d**); at 72 h, mRNA and protein levels of IL-10 were detected by using Q-PCR and ELISA, respectively (**e**). **f** CD4^+^ T cells were treated with anti-CD3/CD28 (2 μg/mL), alpinetin (30 μM), siAhR, CH223191 (10 μM), siAhR + alpinetin, CH223191 + alpinetin, TCDD (5 nM) and siAhR + TCDD for 48 h, expression of miR-302 was detected by using Q-PCR assay. The data were presented as means ± S.E.M. of three independent experiments or six mice in each group. ^##^*P* *<* 0.01 vs. the group without any treatment; ^*^P *<* 0.05 and ^**^P *<* 0.01 vs. DSS group or anti-CD3/CD28 group; ^$$^*P* *<* 0.01 vs. alpinetin (30 μM) group; ^&^*P* *<* 0.05 and ^&&^*P* *<* 0.01 vs. TCDD (5 nM) group

The microRNAs (miRs), highly conserved noncoding single-stranded small RNA molecules, have been shown to play important roles in Treg differentiation, and could regulate the development of immunological diseases^18,19^. The data indicate that AhR activation regulates expressions of miR-31, miR-490, miR-21, miR-155, miR-148a and miR-302^20–24^. Therefore, we investigated whether the miRs participated in alpinetin-induced Treg differentiation after activating AhR. As shown in Fig. 4a, alpinetin (30 mg/kg) significantly promoted expressions of miR-148a and miR-302, but not that of miR-31, miR-490, miR-21 and miR-155. Importantly, the action on miR-302 expression was stronger. The same results were gained from alpinetin (30 μM)-treated CD4^+^ T cells. Notably, TCDD (5 nM) significantly regulated the expressions of miR-148a, miR-302, miR-31, miR-490 and miR-155 in CD4^+^ T cells (Fig. 4b).

Then, to further confirm the participation of miR-302 in alpinetin-improved Treg differentiation, miR-302 mimic and miR-302 inhibitor were used. The miR-302 mimic (50 nM), alpinetin (30 μM) and TCDD (5 nM) showed well promotion of Foxp3 and IL-10 expressions and percentages of Treg cells. In contrast, miR-302 inhibitor (100 nM) obviously restrained the action of alpinetin and TCDD (Fig. 4c–e). Consistently, siAhR and CH223191 (10 μM) inhibited the promotion of miR-302 expression by alpinetin (30 μM) in CD4^+^ T cells, suggesting that alpinetin increased miR-302 expression in an AhR-dependent manner (Fig. 4f).

### The DNMT-1 played a key role in alpinetin-induced Treg differentiation after triggering AhR-miR-302 axis

**Fig. 5:**
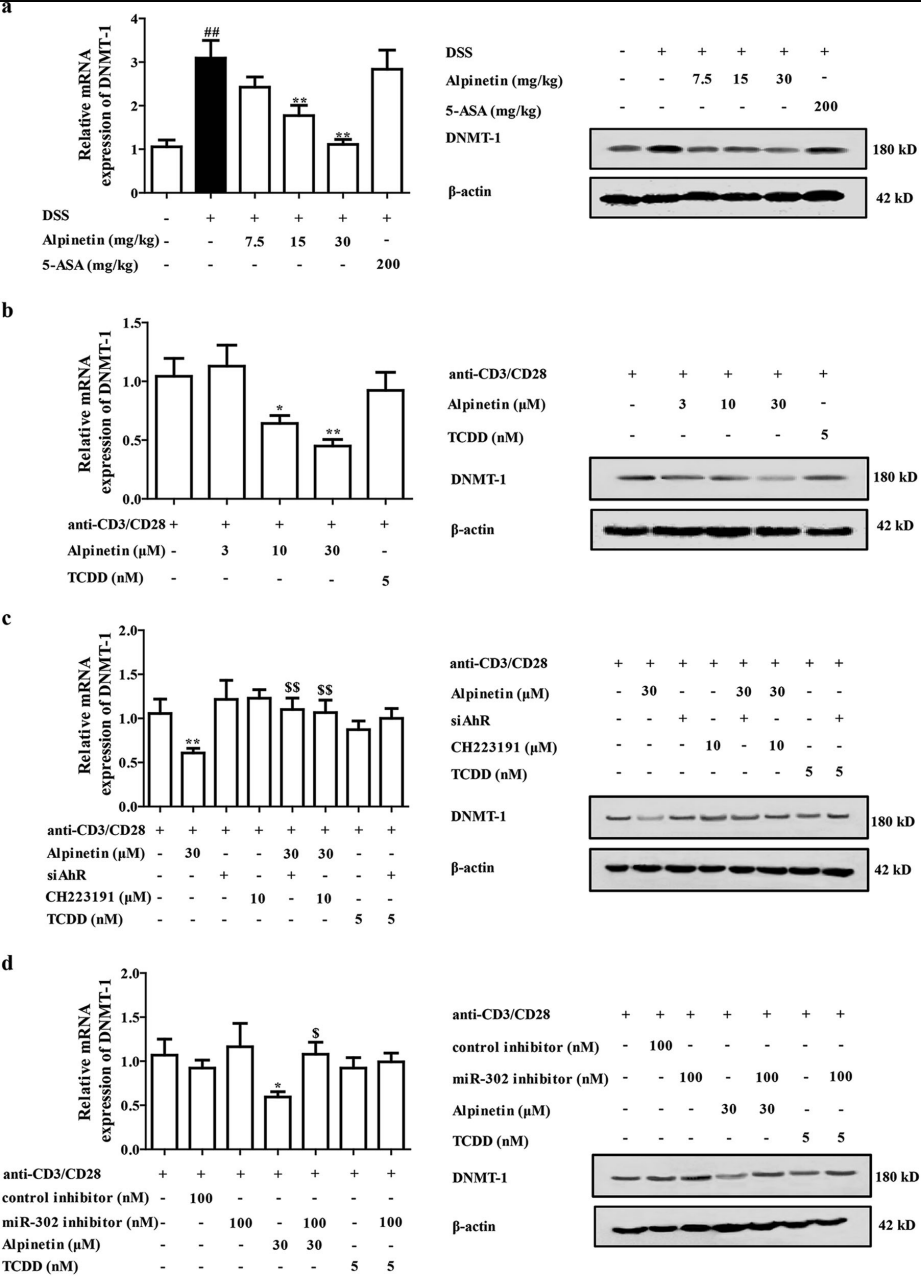

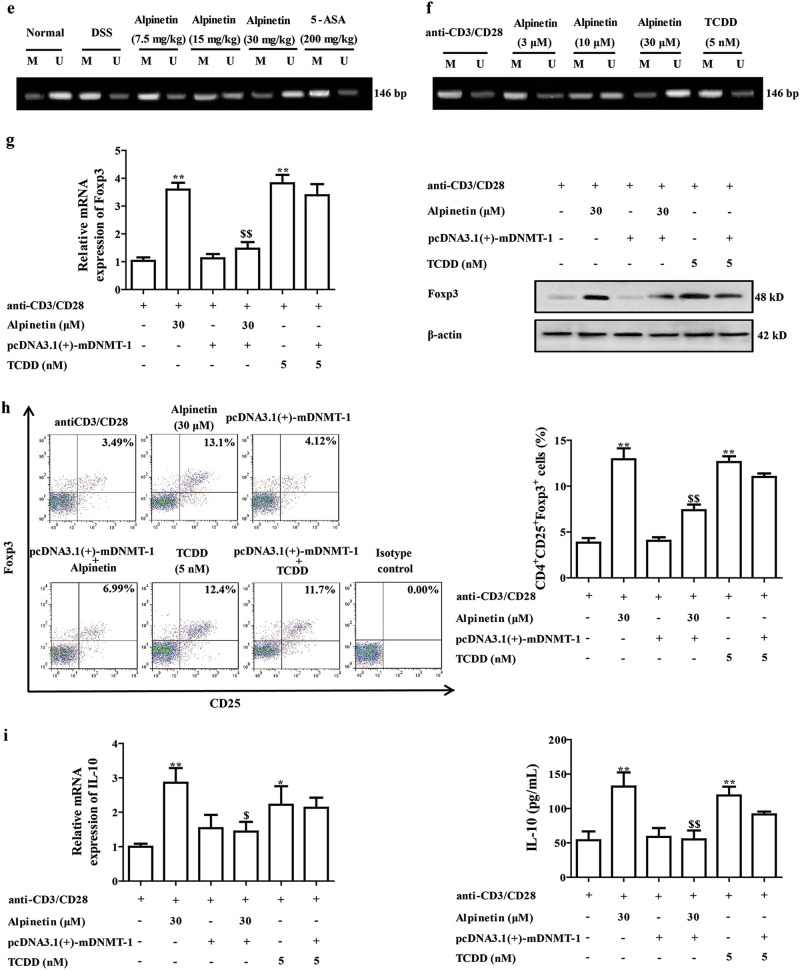
The DNMT-1 participated in alpinetin-promoted Treg differentiation after activating AhR/miR-302 signals. **a** Mice were subjected to colitis, orally administered of alpinetin (7.5, 15, 30 mg/kg) and 5-aminosalicylic acid (5-ASA, 200 mg/kg). Then, colons were isolated, mRNA and protein levels of DNMT-1 were measured by using Q-PCR and western blotting assays, respectively. **b** CD4^+^ T cells were treated with anti-CD3/CD28 (2 μg/mL), alpinetin (3, 10, 30 μM) and TCDD (5 nM) for 48 h. Then, mRNA and protein levels of DNMT-1 were detected by using Q-PCR and western blotting assays, respectively. **c** CD4^+^ T cells were treated with anti-CD3/CD28 (2 μg/mL), alpinetin (30 μM), siAhR, CH223191 (10 μM), siAhR + alpinetin, CH223191 + alpinetin, TCDD (5 nM) and siAhR + TCDD for 48 h. Then, mRNA and protein levels of DNMT-1 were detected by using Q-PCR and western blotting assays, respectively. **d** CD4^+^ T cells were treated with anti-CD3/CD28 (2 μg/mL), alpinetin (30 μM), miR-302 inhibitor (100 nM), miR-302 inhibitor + alpinetin, TCDD (5 nM) and miR-302 inhibitor + TCDD for 48 h. Then, mRNA and protein levels of DNMT-1 were measured by using Q-PCR and western blotting assays, respectively. **e** Mice were subjected to colitis, orally administered of alpinetin (7.5, 15, 30 mg/kg) and 5-ASA (200 mg/kg). On day 10, the colons were collected. The methylation level of Foxp3 promoter region was detected by using methylation-specific PCR assay. **f** CD4^+^ T cells were treated with anti-CD3/CD28 (2 μg/mL), alpinetin (3, 10, 30 μM) and TCDD (5 nM), methylation level of Foxp3 promoter region was detected by methylation-specific PCR assay. **g**–**i** CD4^+^ T cells were treated with anti-CD3/CD28 (2 μg/mL), alpinetin (30 μM), pcDNA3.1(+)-mDNMT-1, pcDNA3.1(+)-mDNMT-1 + alpinetin, TCDD (5 nM) and pcDNA3.1(+)-mDNMT-1 + TCDD for indicated time intervals. At 48 h. mRNA and protein levels of Foxp3 were detected by using Q-PCR and western blotting assays, respectively (**g**); at 72 h, percentages of Treg cells were detected by using flow cytometry assay (**h**); at 72 h, mRNA and protein levels of IL-10 were detected by using Q-PCR and ELISA, respectively (**i**). The data were presented as means ± S.E.M. of three independent experiments or six mice in each group. ^##^*P* *<* 0.01 vs. the group without any treatment; ^*^P *<* 0.05 and ^**^P *<* 0.01 vs. DSS group or anti-CD3/CD28 group; ^$^*P* *<* 0.05 and ^$$^*P* *<* 0.01 vs. alpinetin (30 μM) group

We further explored the deeper mechanism of alpinetin-promoted expression of Foxp3 and subsequent Treg differentiation. Up to now, no information shows the direct relation between miR-302 and Foxp3. Recently, an interesting literature attracts us that DNA methyltransferase 1 (DNMT-1), a candidate target gene of miR-302, possesses the ability to regulate the expression of Foxp3^25,26^. Then, effects of alpinetin and TCDD on DNMT-1 level in colons of colitis mice and CD4^+^ T cells were examined. Results indicated that alpinetin (15, 30 mg/kg) significantly inhibited expression of DNMT-1 in colons of colitis mice. Consistently, alpinetin (10, 30 μM) significantly downregulated expression of DNMT-1 in CD4^+^ T cells, whereas TCDD (5 nM) exerted little effect on level of DNMT-1 in CD4^+^ T cells (Fig. 5a, b). The interesting result of TCDD is beyond our expectation, and the reason was speculated. In the above-mentioned results, TCDD but not alpinetin showed significant elevation of miR-155, and miR-155 could promote expression of DNMT-1 in a sirtuin1-dependent manner. Therefore, TCDD affected both miR-302 (a promoter of DNMT-1) and miR-155 (an inhibitor of DNMT-1), and the effect on DNMT-1 might be slight.

To identify whether alpinetin-inhibited DNMT-1 expression taken place through the induction of AhR activation and miR-302 expression, CD4^+^ T cells were treated with CH223191 (10 μM), siAhR and miR-302 inhibitor (100 nM). As expected, they all significantly recovered alpinetin-inhibited DNMT-1 expression in CD4^+^ T cells (Fig. 5c, d).

DNMT-1 is an enzyme that catalyzes the transfer of methyl groups to specific CpG structures in DNA. It has been demonstrated to play a powerful controlling role in Foxp3 expression^26^. The promoter in the Foxp3 loci has been proposed to be hypomethylated in Treg cells. Demethylation within promoter is not seen in activated T cells that transiently express Foxp3^27^. As shown in Fig. 5e, f, the methylation level of Foxp3 promoter region in colons of colitis mice was inhibited by alpinetin (15, 30 mg/kg) treatment. The same phenomenon was gained in alpinetin (10, 30 μM) but not TCDD (5 nM)-treated CD4^+^ T cells. These data suggested that inhibition of DNA methylation through reduction of DNMT-1 expression in CD4^+^ T cells by alpinetin increases Foxp3 expression and subsequent Treg differentiation.

To further confirm the important role that DNMT-1 played in alpinetin-promoted Treg differentiation, pcDNA(3.1)-mDNMT-1 was prepared and transfected into CD4^+^ T cells. Results showed that pcDNA(3.1)-mDNMT-1 could prevent both alpinetin-promoted expressions of IL-10 and Foxp3 and Treg differentiation (Fig. 5g–i).

### Alpinetin induced Treg differentiation through promoting association of CREB and promoter region of Foxp3 in CD4^+^ T cells after triggering AhR-miR-302-DNMT-1 axis

**Fig. 6:**
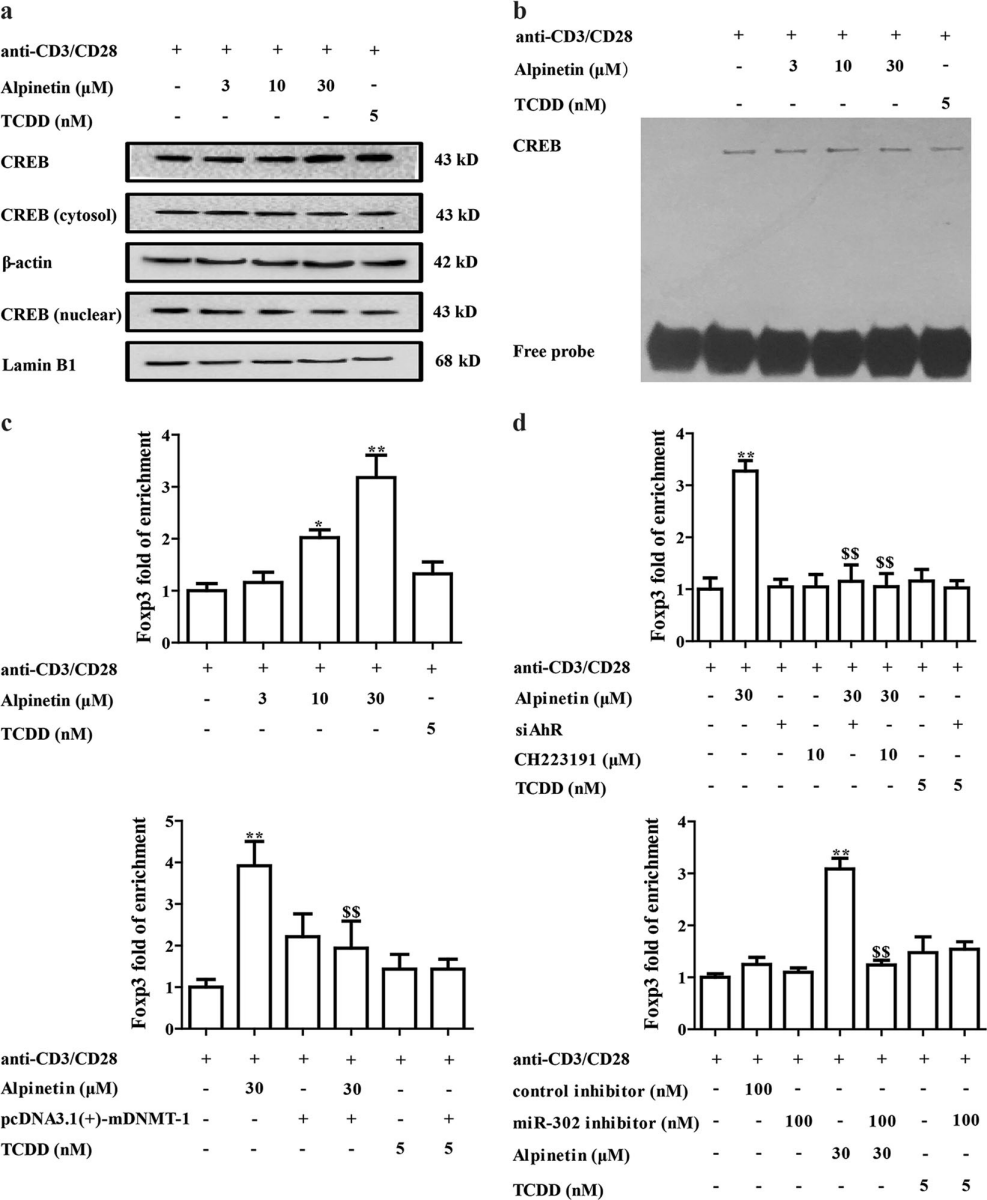
Effect of alpinetin on activation of CREB and association of CREB and de-methylated region of Foxp3 promoter in CD4^+^ T cells. **a**–**c** CD4^+^ T cells were treated with anti-CD3/CD28 (2 μg/mL), alpinetin (3, 10, 30 μM) and TCDD (5 nM) for 48 h. The protein level and nuclear translocation of CREB were detected by using western blotting assay (**a**); the DNA-binding activity of CREB was detected by using EMSA (**b**); the association of CREB and promoter region of Foxp3 was detected by using chromatin-immunoprecipitation (ChIP) assay (**c**). **d** CD4^+^ T cells were treated with anti-CD3/CD28 (2 μg/mL), alpinetin (30 μM), siAhR, CH223191 (10 μM), siAhR + alpinetin, CH223191 + alpinetin, miR-302 (100 nM), miR-302 inhibitor + alpinetin, pcDNA3.1(+)-mDNMT-1 and pcDNA3.1(+)-mDNMT-1 + alpinetin for 48 h. The association of CREB and promoter region of Foxp3 was detected by using ChIP assay. The data were presented as means ± S.E.M. of three independent experiments. ^*^P *<* 0.05 and ^**^P *<* 0.01 vs. anti-CD3/CD28 group; ^$$^*P* *<* 0.01 vs. alpinetin (30 μM) group

The cAMP-response element binding protein (CREB) is one of the best characterized phosphorylation-activated transcription factor in the hasis leucine zipper (bZIP) superfamily. Emerging evidences indicate that CREB can regulate the differentiation and function of Treg cells. It binds with the de-methylated region of Foxp3 promoter and activate its transcriptional activity, eventually inducing the expression of Foxp3^28,29^. To investigate mechanism for alpinetin-enhanced expression of Foxp3 after reducing DNMT-1 expression, we detected the nuclear translocation of CREB, CREB-DNA-binding activity, and association of CREB and Foxp3 promoter region in CD4^+^ T cells. As shown in Fig. 6a, b, alpinetin (10, 30 μM) exerted little effect of nuclear translocation, expression and DNA-binding activity of CREB in CD4^+^ T cells. However, it promoted association of CREB and Foxp3 promoter, which was dependent on AhR, miR-302 and DNMT-1 (Fig. 6c, d). The results demonstrated that alpinetin might not directly drive CREB activation, but improved association of CREB and Foxp3 promoter region by affecting AhR/miR-302/DNMT-1 signals.

### Alpinetin recovered Th17/Treg balance in mice with DSS-induced colitis through regulating AhR/miR-302/DNMT-1/CREB signals

**Fig. 7:**
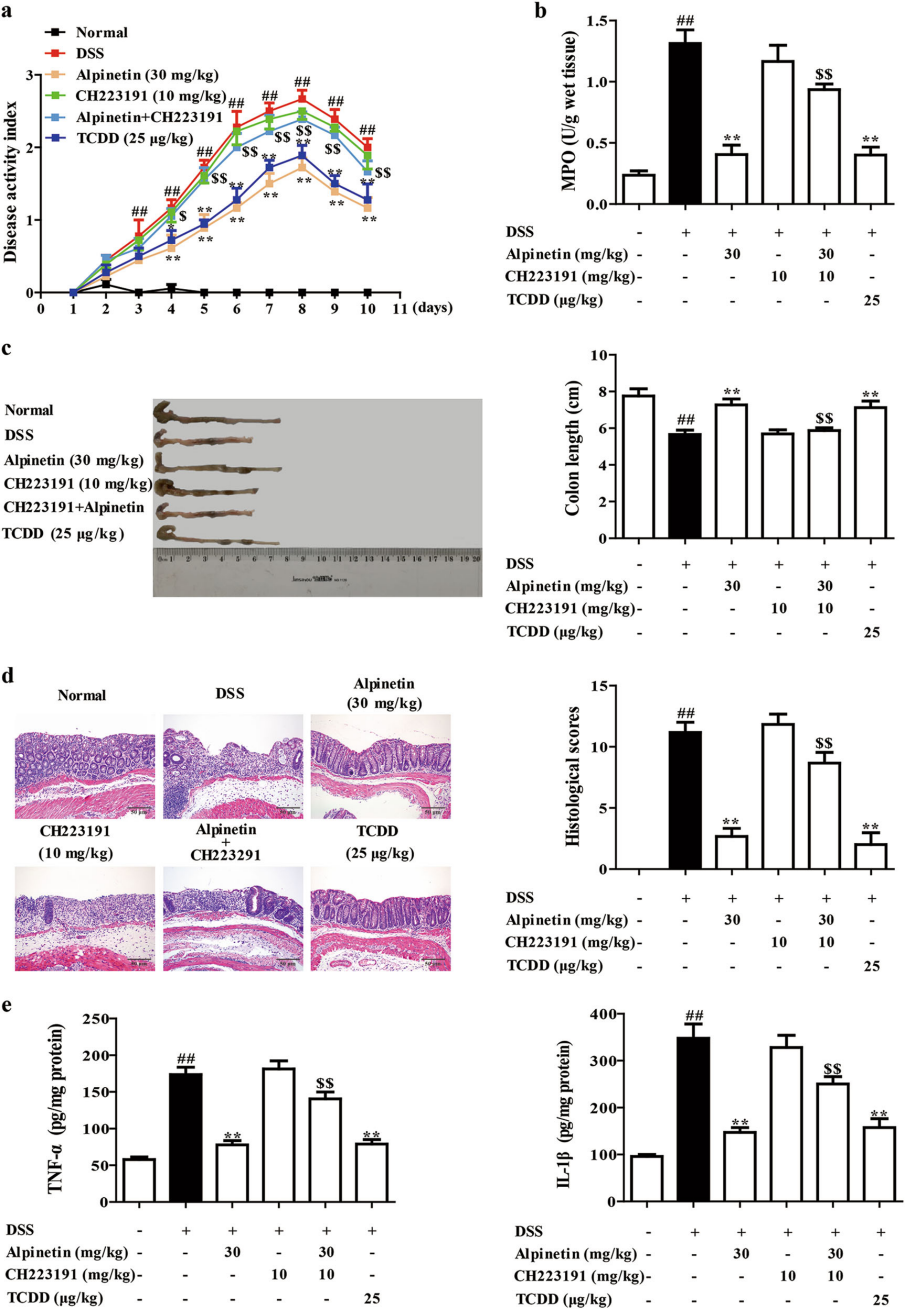
Effect of CH223191 on alpinetin-mediated inhibition of DSS-induced colitis in mice. Mice were subjected to colitis, alpinetin (30 mg/kg, i.g.) and CH223191 (10 mg/kg, i.p.) were administered daily for consecutive 10 days, TCDD (25 μg/kg, i.p.) was administered only at day 1. On day 10, the colons were collected. **a** Disease activity index (DAI) scores. **b** The activity of MPO in colons. **c** The colon length. **d** The histological changes were detected by using hematoxylin and eosin (H&E) staining (Scale bar: 50 μm). **e** The protein levels of TNF-α and IL-1β in colons were detected by using ELISA. Data were presented as means ± S.E.M. of six mice in each group. ^##^*P* < 0.01 vs. the group without any treatment; ^**^P < 0.01 vs. DSS group; ^$^*P* *<* 0.05 and ^$$^*P* *<* 0.01 vs. alpinetin (30 mg/kg) group

**Fig. 8:**
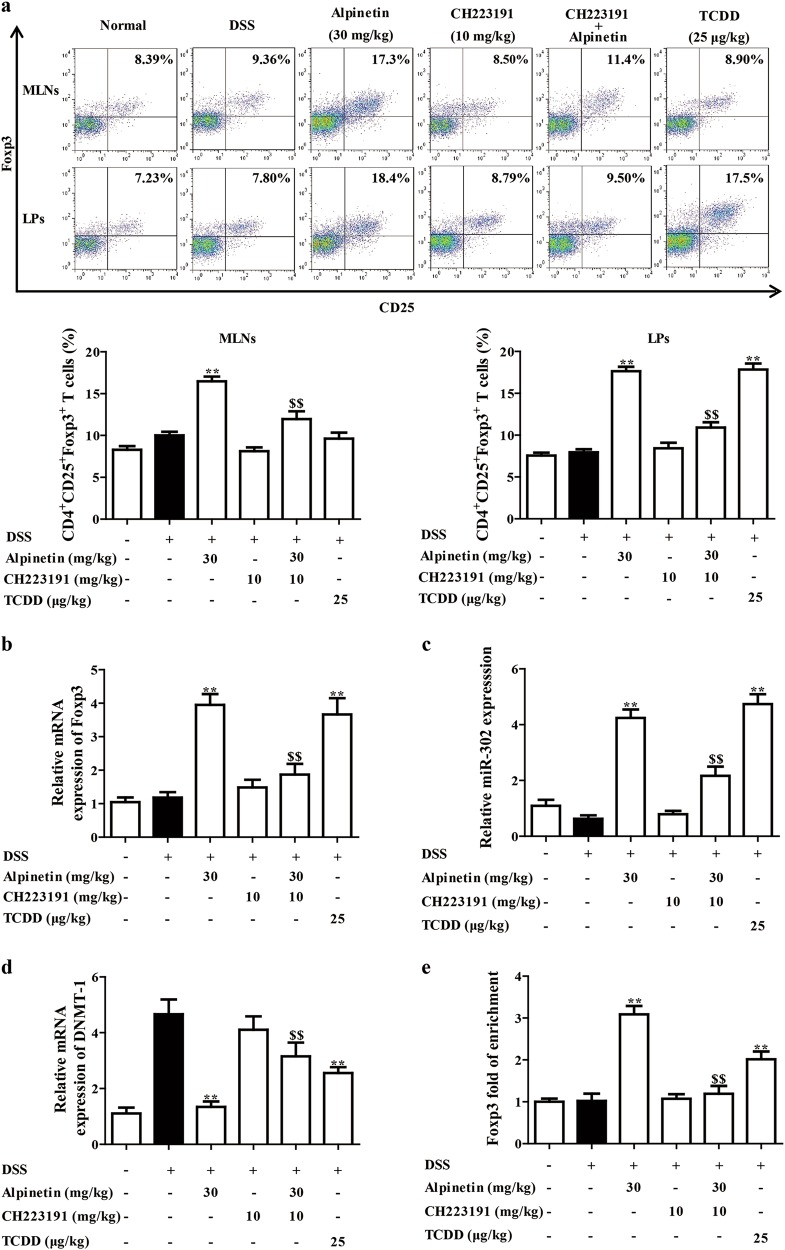
Effect of CH223191 on alpinetin-regulated percentages of Treg cells and miR-302/DNMT-1/CREB signals in colons of mice with DSS-induced colitis. Mice were subjected to colitis, alpinetin (30 mg/kg, i.g.) and CH223191 (10 mg/kg, i.p.) were administered daily for consecutive 10 days. TCDD (25 μg/kg, i.p.) was only administered at day 1. On day 10, the colons and mesenteric lymph nodes (MLNs) were collected. **a** The percentages of Treg cells in MLNs and colonic lamina proprias (LPs) were detected by using flow cytometry assay. **b** The mRNA level of Foxp3 in colons was detected by using Q-PCR assay. **c**, **d** The mRNA levels of miR-302 and DNMT-1 in colons were detected by using Q-PCR assay. **e** The association of CREB and promoter region of Foxp3 in colons was detected by using chromatin-immunoprecipitation (ChIP) assay. The data were presented as the means ± S.E.M. of six mice in each group. ^##^*P* *<* 0.01 vs. the group without any treatment; ^**^P *<* 0.01 vs. DSS group; ^$$^*P* *<* 0.01 vs. alpinetin (30 mg/kg) group

Finally, model of DSS-induced colitis in mice was re-established, CH223191 (10 mg/kg; i.p.), alpinetin (30 mg/kg; i.g.), CH223191 + alpinetin, and TCDD (25 μg/kg; i.p.) were administered. We found that alpinetin (30 mg/kg) showed well reduction of DAI scores, MPO activity, shortening of colon length, histological changes and levels of TNF-α as well as IL-1β in colons of colitis mice, which was restored by CH223191 (10 mg/kg) (Fig. 7a–e). Furthermore, CH223191 prevented alpinetin (30 mg/kg) improved-percentage of Treg cells in MLNs and colonic LPs, and increased-mRNA expression of Foxp3 in colons of colitis mice (Fig. 8a, b).

Then, miR-302/DNMT-1/CREB axis was also investigated. As shown in Fig. 8c–e, alpinetin (30 mg/kg) up-regulated expression of miR-302, downregulated expression of DNMT-1, and promoted association of CREB and promoter region of Foxp3 in colons of colitis mice. However, all the action was prevented by CH223191 (10 mg/kg).

## Discussion

UC is a chronic inflammatory disease mainly occurring at the colon site, which manifests as recurring mucosal inflammation, and spreads from rectum to proximal colon, and even to ileum. Current clinical therapies of UC mainly focus on controlling active inflammation and regulating immune balance, general medicines include 5-ASA, glucocorticoids, immunosuppressive agents and biological agents^30^. However, patients who taking 5-ASA show a high recurrence rate of UC; glucocorticoids and immunosuppressive agents easily lead secondary health problems, and could not be taken as maintenance drugs; biological agents are effective in moderate to severe UC, but are usually with high cost and ineffective for some people^31^. Therefore, new therapeutic agents with high efficiency and few side effects are expected. Our results showed that alpinetin markedly attenuated DSS-induced colitis in mice, and the potency was comparable or even superior to 5-ASA.

Though the pathogenesis of UC is still unclear, the imbalance between Treg and Th17 cells is attractive for the occurrence and development of UC^2^. In the inflamed mucosa and serum of patients with UC, IL-17, and IL-10 levels obviously changed. Depletion of IL-17A markedly ameliorated colitis^4^. In contrast, adoptive transfer of iTreg cells reduces pathological scores and levels of TNF-α and IL-17 in colons of colitis mice, and IL-10-deficient mice spontaneously developed severe colitis^32,33^. CD4^+^ T cells can be induced into Th17 or Treg cells, when the presence of TGF-β and IL-6 or TGF-β alone, respectively. These cells function mainly through secreting IL-17 and IL-10, and are characterized by RORγt and Foxp3, respectively. In UC state, Treg cells can effectively prevent inflammation of colonic mucosal. However, when IL-6 and/or IL-23 exist, Treg cells are transformed into pathogenic Th17 cells^34^. What’s interesting that multiple flavonoids show well regulation of Th17 and Treg differentiation, baicalin promote the differentiation and function of Treg cells induced by TGF-β^15^; epigallocatechin gallate inhibit Th17 differentiation and promote Treg differentiation to prevent experimental autoimmune encephalomyelitis (EAE) and rheumatoid arthritis^35,36^. At present, alpinetin significantly up-regulated the proportions of Treg cells and slightly downregulated that of Th17 cells in MLNs and colonic LPs of colitis mice. In vitro, it concentration-dependently improved the induction of Treg cells but not Th17 cells. In addition, the levels of IL-17, IL-10, Foxp3 and RORγt were similarly regulated both in vivo and in vitro. Taken together, alpinetin could directly affect the generation of Treg cells but not Th17 cells.

AhR, a ligand-dependent receptor, is originally known as the mediator for the environmental toxins (dioxin, TCDD, *etc*) exerted-biochemical and toxic action. In the absence of a ligand, AhR is kept in the cytosol and complexes with the chaperone proteins, including HSP90. Ligand binding results in a conformational change that in turn leads to its nuclear translocation. Release of AhR from its chaperones requires the dimerization of AhR with another bHLH/PAS-domain transcription factor ARNT, which has constitutive nuclear localization. The AhR/ARNT complex binds to XRE to initiate transcription progress of the target genes. In addition, AhR can intervene the differentiation and activation of multiple cells, and regulate the occurrence and development of diseases. The epidemiological data indicate that smoking is negatively related to the incidence and mortality of UC, and the main component of cigarette smoke polycyclic aromatic hydrocarbons (PAH) is a classical AhR agonist. Multiple flavonoids, such as baicalein, luteolin and kaempferol own the ability of activating AhR, and the AhR agonists TCDD and kynurenine could induce the generation of Treg cells^10,11,17^. At present, alpinetin promoted differentiation of Treg cells in an AhR-dependent manner. Furthermore, alpinetin was identified a potential AhR activator, evidenced by induction of CYP1A1 expression and activity, promotion of AhR/HSP90 dissociation and AhR nuclear translocation, induction of XRE reporter activity, and facilitation of AhR/XRE binding. A paper published in nature communication indicate that AhR activation inhibits NLRP3 expression, caspase-1 activation and subsequent IL-1β secretion in peritoneal macrophages, whereas siRNA knockdown of AhR has opposite effects. In addition, He X et al demonstrate that NLRP3 plays important role in alpinetin-ameliorated DSS-induced colitis. All these results further support the opinion that alpinetin might be a potential AhR activator, and it might also prevent the activation of NLRP3 inflammasome *via* AhR^37^.

The miRs, a group of small, endogenous, single-stranded non-coding RNA molecules, act as post-transcriptional negative regulators by directly binding to the 3′-untranslated regions (UTRs) of specific target gene. Recently, data indicate that abnormal expression of miRs can regulate proliferation, apoptosis and differentiation of immune cells^38^. Notably, AhR agonists show regulation of miRs expressions. In naïve T cells, TCDD increase the expression of miR-132/212, when the Th17-polarization condition was provided^39^. In addition, indole-3-carbinol (I3C) and 3, 3′-diindolylmethane (DIM) improve the expression of IL-10 and the percentages of Treg cells in peripheral blood of mice with delayed hypersensitivity reaction by inhibiting expressions of miR-31, miR-219 and miR-490 in guinal lymph node cells^20^; tranilast, an anti-allergy drug, promotes miR-302 expression and cell reprogramming by activating AhR^24^. At present, alpinetin-enhanced expression of miR-302 in colons of colitis mice and CD4^+^ T cells, but not miR-21, miR-155, miR-31, miR-490, and miR-148a. In addition, miR-302 inhibitor significantly abolished alpinetin increased expression of Foxp3 in CD4^+^ T cells and induction of Treg cells. All these results indicated that miR-302 was the key mediator for alpinetin-promoted Treg differentiation after activating AhR.

Up to now, the route of miR-302 walks in regulation of Treg differentiation has not yet been described. The data reveal that its potential downstream target gene might be DNMT-1^25^. The DNA methylation, a common epigenetic modification method, can induce the changes of chromatin structure, DNA conformation, DNA stability and interaction of DNA and protein. The DNA methylation of eukaryotes occurs at the cytosine of CpG dinucleotide, adds methyl groups to fifth carbon atom of cytosine to form 5-methyl cytosine, which is mediates by DNMTs. Data show that level of DNMT-1 in colonic mucosa of UC patients increases with disease activity^40^. In addition, DNMT-1 and DNMT-3b are at a high level in naïve T cells^41^; 5-aza-2’-deoxycytidine (DAC), an DNA methyltransferase inhibitor, can reduce the methylation level of Foxp3 promoter to enhance expression of Foxp3 and induce the generation of Treg cells^42^; DAC increases the proportion of Treg cells in spleens, and subsequently alleviates the disease symptoms of EAE mice^43^. At present, alpinetin markedly downregulated the DNMT-1 expression and methylation level of Foxp3 promoter region in CD4^+^ T cells, while pcDNA3.1(+)-mDNMT-1 showed well prevention of alpinetin-mediated Treg differentiation. Furthermore, siAhR, CH223191 and miR-302 inhibitor also blocked alpinetin-inhibited DNMT-1 expression. Additionally, methylation of the CpG island in Foxp3 promoter inhibits the binding of CREB and decreases Foxp3 gene expression. Conversely, decreased methylation of the CpG island in Foxp3 promoter induces the binding of CREB and increases the gene expression of Foxp3^28,29^. At present, alpinetin showed little effect of nuclear translocation, expression and DNA-binding activity of CREB, but significantly promoted the association of CREB and promoter region of Foxp3, which was prevented by siAhR, CH223191, miR-302 inhibitor and pcDNA3.1(+)-mDNMT-1. Finally, colitis was induced by DSS in mice, and alpinetin (30 mg/kg) was administered in combination with CH223191 (10 mg/kg). CH223191 almost reversed alpinetin-alleviated UC symptoms, promoted proportion of Treg cells, and the following signals.

In summary, alpinetin significantly ameliorated colitis in mice by recovering Th17/Treg balance. The deep mechanisms might be summed up as: activating AhR, promoting expression of miR-302, downregulating expression of DNMT-1, reducing methylation level of Foxp3 promoter region, facilitating combination of CREB and promoter region of Foxp3, and up-regulating the expression of Foxp3.

## Materials and methods

### Chemicals and reagents

Alpinetin (C_16_H_14_O_4_, MW: 270.28, purity >98%) was purchased from Jingzhu biological technology (Nanjing, China)^44^; 5-ASA sustained release granules were purchased from Ipsen Pharma (Houdan, France); TCDD was purchased from J&K Chemical (Beijing, China); CH223191 was purchased from Selleckchem (Houston, USA); fetal bovine serum (FBS) was purchased from PAA (Linz, Germany); DSS (36–50 kDa) was purchased from MP Biomedical (Aurora, USA); MPO activity detection kit was purchased from Nanjing Jiancheng Bioengineering Institute (Nanjing, China); nuclear and cytoplasmic protein extraction kit were purchased from Nanjing KeyGen Biotech. Inc. (Nanjing, China); IL-1β, TNF-α, IL-10 and IL-17A enzyme-linked immune sorbent assay (ELISA) kits were purchased from Dakewe Biotech (Shenzhen, China); FITC-anti-CD4, APC-anti-CD25, PE-anti-Foxp3, PE-anti-IL-17A, IgG2a K isotype control PE, fixation/permeabilization concentrate, diluent reagent, purified anti-mouse CD3e and anti-mouse CD28 mAbs were purchased from eBioscience (San Diego, USA); PMA/Ionomycin mixture and BFA/Monensin mixture were purchased from MultiSciences Biotech (Hangzhou, China); Mouse CD4^+^ CD62L^+^ T-cell isolation kit II was purchased from Miltenyi Biotech (Cologne, Germany); rmIL-2, rmIL-6 and rhTGF-β1 were purchased from PeproTech (Madison, USA); MTT, lipofectamine ^TM^ 2000 and TRIzol reagent were purchased from Invitrogen (Carlsbad, USA); anti-CYP1A1 antibody, anti-β-actin antibody, anti-lamin B antibody, protein A + G agarose beads, peroxidase-conjugated secondary antibody and pcDNA3.1(+)-mDNMT-1 were purchased from Bioworld (Georgia, USA); siAhR was purchased from Genechem (Shanghai, China); the Bulgen-loop^TM^ miRNA qRT-PCR Primer Sets specific for miR-31, miR-219, miR-302, miR-148a, miR-21 and miR-155 were purchased from RiboBio (Guangzhou, China); EROD activity detection kit was purchased from Shanghai genmed Pharmaceutical Technology Co. Ltd. (Shanghai, China); HiScript™QRTSuperMix and AceQ™qPCR SYBR^®^ Green Master Mix were purchased from Vazyme Biotech (Piscataway, USA); EZ DNA Methylation-Gold™ Kit was purchased from Zymo Research Corporation (CA, USA); genomic DNA Mini Preparation Kit, ChIP assay Kit, proteinase K and NP40 buffer were purchased from Beyotime Biotech (Nanjing, China); anti-DNMT-1 antibody was purchased from Abcam (Cambridge, UK); anti-CREB antibody was purchased from Cell Signaling Technology (MA, USA); anti-Foxp3 antibody, anti-AhR antibody, anti-HSP90 antibody and anti-ARNT antibody were purchased from Santa Cruz Biotechnology (CA, USA); ECL reagent was purchased from DiZhao Biotech (Shanghai, China).

In vivo, TCDD and CH223191 were diluted in corn oil; alpinetin and 5-ASA were diluted in 0.5% carboxymethyl cellulose sodium-Na (CMC-Na). In vitro, alpinetin and CH223191 were firstly dissolved in dimethylsulfoxide (DMSO) for stock solution at 1 M, which were further diluted to the required concentration (alpinetin: 1, 3, 10, 30 μM; CH223191: 10 μM) by using serum-free medium. Notably, the final DMSO concentration in the solutions of alpinetin and CH223191 is far less than 0.1% (v/v). In addition, the purchased TCDD solution dissolved in isooctane (10 μg/mL) was directly diluted to the required concentration (5 nM) by using serum-free medium, the final isooctane concentration in the solution of TCDD is far less than 0.1% (v/v).

### Animals

Female C57BL/6 mice (8–10 weeks), weighting 20–22 g, were purchased from the Comparative Medicine Centre of Yangzhou University (Yangzhou, China). All mice were maintained in plastic cages (290 × 178 × 160 mm) with free access to food and water, and housed at 22 ± 1 °C with a 12 light/dark cycle. The animal experiments were conducted with the approval of the Animal Ethics Committee of China Pharmaceutical University, and conformed to the National Institute of the Health guidelines on the ethical use of animals.

### Induction of UC and treatment

Mice were fed with 2.5% DSS (dissolved in sterile distilled water) for 7 days, and followed by sterile distilled water alone for another 3 days. They were randomly divided into the following groups: (a) To identify the anti-colitis action of alpinetin: Normal group, DSS group, alpinetin (7.5, 15, 30 mg/kg) groups and 5-ASA (200 mg/kg) group; (b) To identify the key role of AhR played in the anti-colitis action of alpinetin: Normal group, DSS group, alpinetin (30 mg/kg) group, CH223191 (10 mg/kg) group, CH223191 + alpinetin group and TCDD (25 μg/kg) group. The alpinetin and 5-ASA were orally administered from day 1 to 10; TCDD was intraperitoneally administered only on day 1; CH223191 was intraperitoneally administered from day 1 to 10. In addition, mice in Normal and DSS groups were given an equal volume of vehicle.

### DAI scores

From day 1 to 10, body weight loss, stool consistency and gross bleeding of each mice were recorded. DAI scores were calculated as the mean value: (a) body weight loss: 0 = none; 1 = 1–5%; 2 = 5–10%; 3 = 10–20%; 4 = over 20%; (b) stool consistency: 0 = normal; 2 = loose stools; 4 = diarrhea; (c) gross bleeding: 0 = negative; 2 = positive; 4 = gross rectal bleeding.

### Histopathological examination

On day 10, mice were executed. The colons were collected and photographed, and the length was measured. Then, they were fixed in 10% phosphate-buffered saline (PBS)-buffered formalin, and stained with H&E for histological examination. The histological scores were graded as follows: (a) the severity of inflammation: 0 = none; 1 = mild; 2 = moderate; 3 = severe; (b) the lesion depth: 0 = none; 1 = mucosal layer; 2 = submucosal layer; 3 = muscle layer; 4 = transmural; (c) crypt damage: 0 = none; 1 = basal 1/3 damaged; 2 = basal 2/3 damaged; 3 = only surface epithelium intact; 4 = entire crypt and epithelium lost; (d) lesion range: 1 = 1–25%; 2 = 26–50%; 3 = 51–75%; 4 = 76–100%. The scores were calculated by adding the 3 evaluations and giving a maximal score of 10.

### MPO activity

On day 10, the colons were collected. Then, they were homogenized with normal saline, centrifuged at 3000 rpm for 5 min. The supernatant was collected, and MPO activity was measured by using a kit according to the manufacturer’s instructions^45,46^.

### ELISA

On day 10, colons were collected. Then, they were homogenized with normal saline, centrifuged at 3000 r.p.m. for 5 min. The supernatant was collected, levels of IL-1β, TNF-α, IL-10, and IL-17 were detected by using ELISA kits according to the manufacturer’s instructions.

### Q-PCR assay

On day 10, colons were collected, mRNA levels of Foxp3, RORγt, IL-17, IL-10 and CYP1A1 were detected;

EL-4 cells were seeded into 6-well plates at a density of 1 × 10^6^ cells/mL, treated with alpinetin (3, 10, 30 μM) and TCDD (5 nM) for 24 h, and mRNA level of CYP1A1 was detected;

CD4^+^ T cells were seeded into 6-well plates at a density of 1 × 10^6^ cells/mL, treated with anti-CD3/CD28 (2 μg/mL), alpinetin (3, 10, 30 μM) and TCDD (5 nM) for 48 h, and mRNA levels of Foxp3 and DNMT-1 were detected.

The total RNA in colons or cells was extracted by using TRIzol extraction reagent. The purity and concentration of total RNA was determined by measuring the absorbance value at 260 and 280 nm. The complementary DNA (cDNA) was reversely transcribed from 1 μg total RNA by using HiScript™QRTSuperMix, and Q-PCR was carried out by using AceQ™qPCR SYBR^®^ Green Master Mix in a CFX96 ^TM^ Real-Time PCR Detection Systems (Bio-Rad, Hercules, CA, USA). The primer sequences were as follows: GAPDH forward: 5′-GACATTTGAGAAGGGCCACAT-3′, reverse: 5′-CAAA GAGGTCCAAAACAATCG-3′; Foxp3 forward: 5′-GCCCATCCAATAAACTGTGG-3′, reverse: 5′-GTATCCGCTTTCTCCTGCTG-3′; RORγt forward: 5′-CCCTCTGGCACACAATCTCT-3′, reverse: 5′-CGGTCCTCTGCTTCTCTTAGG-3′; IL-17 forward: 5′-AGACAAAGCCAGAGTCCTTCAG-3′, reverse: 5′-TTAGGAGAGCATTGGAAATTGG-3′; IL-10 forward: 5′-GCCTTATCGGAAATGATCCA-3′, reverse: 5′-AGGGTCTTCAGCTTCTCACC-3′; CYP1A1 forward: 5′-TCTGTGCCATTTGCTTTGGC-3′, reverse: 5′-AGGCATTCAGGGAAGGGTTG-3′; DNMT-1 forward: 5′-TGGTGTTGTCTACCGACTGG-3′, reverse: 5′-CAGGGTCTCGTTCACAGGAT-3′.

### Isolation of lymphocytes in MLNs and colonic LPs

On day 10, MLNs were collected and mechanically dissociated. The single cell suspensions were filtered by using nylon membrane and centrifuged at 1200 rpm for 5 min. The precipitation was re-suspended with RPMI 1640 supplemented with 10% FBS, and used for flow cytometry assay.

On day 10, colons were collected. They were cut into 1 cm segments, and incubated with RPMI 1640 supplemented with 1 mM EDTA, 1 mM DTT, 10% FBS and 50 μg/mL gentamycin for 40 min at 37 °C to remove epithelial cells. The residuary parts were mechanically dissociated with PBS, filtered by using nylon membrane, and centrifuged at 1200 r.p.m. for 20 min. The lymphocytes were isolated by using separation medium, and used for flow cytometry assay.

### Cell culture

Naive CD4^+^ T cells from MLNs of C57BL/6 mice were purified with magnetic beads (CD4^+^ CD62L^+^ T Cell Isolation Kit II, Miltenyi Biotech). Briefly, C57BL/6 mice were sacrificed and the MLNs were collected, followed by grinding with 1 mL phosphate buffer saline (PBS) in a glass homogenizer until cell suspensions were obtained. Subsequently, the cell suspensions were collected and centrifuged at 1200 rpm for 5 min. The precipitates were retained and suspended in 400 μL PBS, followed by addition of 100 μL of biotin-antibody cocktail. The mixture was then blended and incubated for 15 min at 4 °C. Afterwards, 300 μL buffer and 200 μL anti-biotin microbeads were added and mixed, followed by incubation for 10 min at 4 °C. Finally, the mixture was flown into MACS-LD column (Miltenyi Biotech, Cologne, Germany), and the filtrate was collected. The filtrate was centrifuged at 1200 rpm for 5 min and re-suspend in RPMI 1640 medium supplemented with 100 U/mL of streptomycin, 100 U/mL of penicillin and 10% fetal calf serum under a humidified 5% (v/v) CO_2_ atmosphere at 37 °C.

### Treg and Th17 differentiation

Data indicated that classical AhR agonists, including TCDD, DIM and I3C, can directly induce the differentiation of Treg cells in vitro without TGF-β stimulation. Considering that alpinetin might be a potential AhR activator, effect of alpinetin on Treg differentiation was carried out in the absence of TGF-β stimulation. CD4^+^ T cells were treated with anti-CD3/CD28 (2 μg/mL) and alpinetin (1, 3, 10, 30 μM) for 72 h, the frequencies of Treg cells were detected by using flow cytometry assay^8,10,20,47^.

For Th17 differentiation, CD4^+^ T cells were treated with anti-CD3/CD28 (2 μg/mL), rhTGF-β1 (5 ng/mL), rmIL-6 (20 ng/mL), rmIL-2 (300 IU/mL) and alpinetin (1, 3, 10, 30 μM) for 72 h, the frequencies of Th17 cells were detected by using flow cytometry assay.

### Flow cytometry assay

Detection of Treg cells: lymphocytes and CD4^+^ T cells were incubated with FITC-anti-CD4 and APC-anti-CD25 for 30 min. After being fixated and permeabilizated, cells were further stained with PE-anti-Foxp3 for 1 h. The flow cytometry was conducted on a FACS Calibur (Becton Dickinson, New York, USA), and the data were analyzed by using FlowJo7.6.1 software.

Detection of Th17 cells: lymphocytes and CD4^+^ T cells were stimulated with PMA/Ionomycin mixture and BFA/Monensin mixture for 5 h, and incubated with FITC-anti-CD4 for 30 min. After being fixated and permeabilizated, cells were stained with APC-anti-IL-17 for 1 h. The flow cytometry was conducted on a FACS Calibur (Becton Dickinson, New York, USA), and data were analyzed by using FlowJo7.6.1 software.

### Detection of cell viability and proliferation

The viability of CD4^+^ T cells or EL-4 cells was evaluated by using MTT assay. Briefly, they were seeded into 96-well plates at a density of 1 × 10^6^ cells/mL, and treated with alpinetin (1, 3, 10, 30, 100 μM) for 68 h. 20 μL of MTT solution (5 mg/mL dissolved in PBS) was added into each well for another 4 h. Finally, the supernatant was removed, and 150 μL DMSO was added to each well to dissolve the formazan crystals. The absorbance was measured at 570 nm.

The proliferation of CD4^+^ T cells was evaluated by using CCK-8 assay. Briefly, they were seeded into 96-well plates at a density of 1 × 10^6^ cells/mL, and treated with alpinetin (1, 3, 10, 30, 100 μM) for 68 h. 10 μL of CCK-8 solution was added into each well for another 4 h. The absorbance was measured at 450 nm.

### EROD activity

EL-4 cells were seeded into 6-well plates at a density of 1 × 10^6^ cells/mL, and treated with alpinetin (3, 10, 30 μM), siAhR, CH223191 (10 μM), siAhR + alpinetin (30 μM), CH223191 + alpinetin (30 μM) and TCDD (5 nM) for 24 h, and supernatant was collected. Then, activity of CYP1A1 was detected by using an EROD enzyme kit, fluorescence intensity was measured by using a FL600 plate reader with excitation at 530 nm and emission at 590 nm (Biotek, Winooski, VT, USA).

### Transfection

Stable transfection: EL-4 cells and CD4^+^ T cells were seeded into 6-well plates at a density of 1 × 10^6^ cells/mL, and transfected with lentivirus-mediated siAhR for 72 h according to the manufacturer’s instructions^48^.

Transient transfection: CD4^+^ T cells were seeded into 6-well plates at a density of 1 × 10^6^ cells/mL, and transfected with pcDNA3.1-(+)-mDNMT-1, miR-302 mimic, miR-302 inhibitor by using lipofectamine^TM^ 2000 reagent for 24 h according to the manufacturer’s instructions.

### Western blotting

On day 10, colons were collected, and protein levels of CYP1A1 and DNMT-1 were detected;

EL-4 cells were seeded into 6-well plates at a density of 1 × 10^6^ cells/mL, and treated with alpinetin (3, 10, 30 μM), Act-D (5 μg/mL), Act-D + alpinetin (30 μM) and TCDD (5 nM) for 24 h, protein level of CYP1A1 was detected;

CD4^+^ T cells were seeded into 6-well plates at a density of 1 × 10^6^ cells/mL, and treated with a): anti-CD3/CD28 (2 μg/mL), alpinetin (3, 10, 30 μM), miR-302 mimic (50 nM), miR-302 inhibitor (100 nM), miR-302 inhibitor + alpinetin (30 μM), TCDD (5 nM) and miR-302 inhibitor + TCDD, and protein levels of Foxp3, IL-10 and DNMT-1 were detected; b) anti-CD3/CD28 (2 μg/mL), alpinetin (30 μM), pcDNA3.1(+)-mDNMT-1, pcDNA3.1(+)-mDNMT-1 + alpinetin, TCDD (5 nM) and pcDNA3.1( + )-mDNMT-1 + TCDD, and protein level of Foxp3 and IL-10 were detected; c) anti-CD3/CD28 (2 μg/mL), alpinetin (30 μM), siAhR, CH223191 (10 μM), siAhR + alpinetin, CH223191 + alpinetin, TCDD (5 nM) and siAhR + TCDD, and protein level of DNMT-1 was detected; d) anti-CD3/CD28 (2 μg/mL), alpinetin (3, 10, 30 μM) and TCDD (5 nM), and protein level of CREB was detected.

The protein was extracted by using NP-40 lysis buffer or a nuclear and cytoplasmic protein extraction kit, and quantified by using bradford assay. Subsequently, they was separated on 10% SDS-PAGE gel, and transferred to polyvinylidene fluoride (PVDF) membranes. The membranes were blocked with 9% non-fat-milk for 2 h at room temperature, incubated with specific primary antibodies at 4 °C for overnight, and washed with TBST for 3 times. Then, they were incubated with HRP-conjugated secondary antibody at 37 °C for 2 h. Finally, the protein bands were visualized by using enhanced chemiluminescent (ECL) reagent.

### HPLC assay

EL-4 cells were seeded into cell culture bottles at a density of 1 × 10^6^ cells/mL, and treated with alpinetin (30 μM) for 0.5, 1, 2, 3 and 4 h. Then, cells were collected, centrifuged at 1200 r.p.m. for 5 min, repeated freezing and thawing 5 times. Then, they were centrifuged at 12,000 r.p.m. for 5 min, supernatant was collected. Subsequently, the liquid was dried, methanol was added to dissolve each sample, and alpinetin was detected by using HPLC assay. The chromatographic separation was achieved with a Hedera ODS-2 C18 column (150 mm × 2.1 mm; 5 μm; Hanbon, Jiangsu, China) at 30°C. The mobile phase consisted of solvent A (methanol) and solvent B (water) (70: 30, v/v) at a flow rate of 1 mL/min, and detection wave length was set at 300 nm.

### Co-immunoprecipitation (Co-IP) assay

EL-4 cells were seeded into cell culture bottles at a density of 1 × 10^6^ cells/mL, and treated with alpinetin (30 μM), CH223191 (10 μM), CH223191 + alpinetin and TCDD (5 nM) for 24 h, accumulation of AhR/HSP90 and AhR/ARNT complexes was detected.

The proteins were isolated from EL-4 cells by using stronger RIPA lysis buffer, incubated with 1 μg antibody against HSP90 or ARNT at 4 °C for overnight, followed by addition of 20 μL protein A + G agarose. Moreover, they were rotated at room temperature for 4 h. Immunoprecipitates were washed with RIPA lysis buffer for 4 times, and western blotting was performed as previously described.

### Immunofluorescence assay

EL-4 cells were seeded into 6-well plates at a density of 1 × 10^6^ cells/mL, and treated with alpinetin (30 μM) and TCDD (5 nM) for 24 h, nuclear translocation of AhR was detected.

The cells were fixed with 4% paraformaldehyde, and permeabilized with 0.5% Triton X-100 for 20 min. Subsequently, they were blocked with 5% bovine serum albumin (BSA) for 2 h, and incubated with antibody against AhR at 4 °C for overnight. After being washed with PBS for 3 times, cells were stained with specific IgG antibody for 2 h. Finally, the coverslips were stained with DAPI for additional 20 min, and images were obtained under a fluorescence microscope.

### Electrophoretic mobility shift assay (EMSA)

EL-4 cells were seeded into cell culture bottles at a density of 1 × 10^6^ cells/mL, and treated with alpinetin (30 μM), siAhR, CH223191 (10 μM), siAhR + alpinetin, CH223191 + alpinetin and TCDD (5 nM) for 24 h, DNA-binding activity of AhR was detected. The nuclear proteins were isolated, and DNA-binding activity of AhR/ARNT/xenobiotic response elements (XRE) was detected by using an EMSA kit according to the manufacturer’s instructions (Pierce; Rockford, IL, USA). A complementary pair of synthetic oligonucleotides containing the XRE binding site for the transformed AhR/ARNT complexes (5′-GATCTGGCTCTTCTCACGCAACTCCG-3′ and 5′-GATCCGGAGTTGCGTGAGAAGAGCCA-3′) were synthesized, purified, and labeled with biotin. The nuclear extracts were mixed with poly (dI-dC), labeled probe, binding buffer and incubated for 10 min. Subsequently, protein-DNA complex (10 μL) was fractionated on a gel electrophoresis at 10 V/cm for 1 h at 4 °C. Then, they were transferred to a nylon membrane. For supershift assay, the nuclear extracts were incubated with anti-AhR antibody for 30 min at 37 °C before the complexes were analyzed by EMSA. The bands on nylon membranes were visualized by using film exposure with ECL reagent.

### Luciferase reporter assay

EL-4 cells (1 × 10^6^ cells/mL) were seeded into 6-well plates and incubated with pGL3-XRE reporter gene plasmid by using lipofectamine ^TM^ 2000 reagent for 24 h. Afterwards, the cells were exposed to alpinetin (30 μM) in the presence or absence of CH223191 (10 μM) for 24 h. The luciferase activity was measured by using a luciferase assay system and a multimode reader^49^.

### DNA preparation and Methylation-specific PCR (MSP)

On day 10, colons were collected, and methylation level of Foxp3 promoter region was detected;

CD4^+^ T cells were treated with anti-CD3/CD28 (2 μg/mL), alpinetin (3, 10, 30 μM) and TCDD (5 nM), methylation level of Foxp3 promoter region was detected.

Total DNA was extracted from colons or cells by using a genomic DNA mini preparation kit according to the manufacturer’s instructions. The purity and concentration was quantified by measuring absorbance at 260 and 280 nm. The promoter region of Foxp3 gene was checked through the website (http://genome.ucsc.edu/), which was further put into methprimer (http://www.urogene.org/cgi-bin/methprimer/methprimer_results.cgi) to determine the MSP primer sequences. Corresponding methylated (M) and un-methylated (U) primers to each CpG site were designed and synthesized by Sangon Biotech Corporation (Shanghai, China). The bisulfite conversion of genomic DNA, used to investigate un-methylated *versus* methylated cytokines, was conducted by using EZ DNA Methylation-Gold™ Kit as the manufacturer’s instructions.

### Chromatin-immunoprecipitation (ChIP) assay

On day 10, colons were collected, and association of CREB and promoter region of Foxp3 was detected;

CD4^+^ T cells were treated with a): anti-CD3/CD28 (2 μg/mL), alpinetin (3, 10, 30 μM), TCDD (5 nM); b): anti-CD3/CD28 (2 μg/mL), alpinetin (30 μM), siAhR, CH223191 (10 μM), siAhR + alpinetin, CH223191 + alpinetin, TCDD (5 nM), siAhR + TCDD; c): anti-CD3/CD28 (2 μg/mL), alpinetin (30 μM), miR-302 inhibitor, miR-302 inhibitor + alpinetin, TCDD (5 nM), miR-302 inhibitor + TCDD; d): anti-CD3/CD28 (2 μg/mL), alpinetin (30 μM), pcDNA3.1( + )-mDNMT-1, pcDNA3.1( + )-mDNMT-1 + alpinetin, TCDD (5 nM), pcDNA3.1( + )-mDNMT-1 + TCDD for 48 h, and association of CREB and promoter region of Foxp3 was detected.

The ChIP assay was performed by using a commercial kit according to the manufacturer’s protocols. Briefly, CD4^+^ T cells were incubated with 1% formaldehyde at 37 °C for 10 min to allow the cross-linking of endogenous proteins and DNA. After being washed with PBS supplemented with 1 mM PMSF, cells were re-suspended with buffer containing 1% SDS and 1 mM PMSF and lysed by using a sonicator (Scientz-IID, China). Then, samples were centrifuged at 5000 rpm for 10 min, and the supernatant was collected. The chromatin in supernatant was immunoprecipitated with antibody against CREB or IgG. After immunoprecipitation, the protein-DNA crosslinks were reversed by heating at 65 °C, followed by proteinase K treatment. DNA was purified by using the DNA purification kit (Beyotime Biotech, Nanjing, China) and then amplified by using Q-PCR as previously described.

### Statistical analysis

Statistical analysis was performed with SPSS statistical software (SPSS, Chicago, IL, USA), and data were expressed as means ± S.E.M. The mean differences between two groups were compared by *t* test; the mean differences between multiple groups were compared by one-way ANOVA and Fisher’s Least Significant Difference (LSD) test. A value of *P* less than 0.05 (*P* < 0.05) was accepted as a significant difference.
